# Improved Research on Two-Step Thermal Stress Calculation Method for Asphalt Mixture: Extended Creep Compliance Test

**DOI:** 10.3390/ma17122939

**Published:** 2024-06-15

**Authors:** Xu He, Peng Li, Bo Lin, Shuangquan Jiang

**Affiliations:** 1 School of Highway, Chang’an University, Xi’an 710064, China; lipeng2013@chd.edu.cn (P.L.); jiangsq5311@126.com (S.J.); 2 Department of Civil Architectural and Environmental Engineering, Missouri University of Science and Technology, Rolla, MO 65401, USA; bolin@mst.edu

**Keywords:** thermal stress, cracking temperature, extended creep compliance test, cooling rate, initial temperature

## Abstract

The two-step thermal stress calculation method (TTSCM) is commonly used to predict the cracking temperature of asphalt mixture. The aim of this study is to improve TTSCM’s mathematical model so as to enhance its prediction accuracy. First, this study evaluated the errors of predicted cracking temperatures of original TTSCM for AC-16 and AC-25 asphalt mixtures by thermal stress-restrained specimen test (TSRST). Then, an improved method called the extended creep compliance test (ECCT) was developed to modify the TTSCM. The test results show that the cracking predictions of the original TTSCM are not always accurate. Particularly for AC-16 asphalt mixture, the predicted cracking temperature is 2.9 °C (−10.6%) higher than the measured value by the TSRST. The ECCT method has been proven to be an effective way to enhance the prediction accuracy of the TTSCM. The predicted cracking temperatures modified by the ECCT method for both asphalt mixtures are relatively accurate, having an error within ±2%. The ECCT method changed the calculated thermal stress values at different temperatures of the TTSCM; however, they still conformed to a basic changing trend with respect to the initial temperature and cooling rate. Finally, a recommendation regarding the ECCT method was presented.

## 1. Introduction

Low-temperature cracking is a prevalent distress in asphalt pavement, particularly in cold climates [1]. As the temperature decreases, the asphalt mixture gradually transitions to a brittle state, accompanied by an increase in thermal stress. Furthermore, as the temperature reaches a critical threshold (commonly referred to as the cracking temperature), the thermal stresses in asphalt mixtures exceed their tensile strength. At this time, low-temperature cracking occurs, usually resulting in transverse cracks [2,3,4]. Notably, low-temperature cracking can facilitate the infiltration of surface water into a pavement structure [5], thereby compromising its load-bearing capacity. This contributes to a reduction in the service life of asphalt pavement [6].

The two-step thermal stress calculation method (TTSCM) [7] is commonly used to predict the cracking temperatures of asphalt mixtures. This method is dependent on a simple linear viscoelastic model and is divided into two steps. First, the creep compliance, D(t), of the asphalt mixture obtained from the creep test is converted to the relaxation modulus, E(t), and the relaxation modulus master curve is then developed and fitted using a mathematical model [8]. Second, the thermal stress of the asphalt mixture is derived using the E(t) convolution integral [5,9,10]. The cracking temperature of the asphalt mixture is predicted by intersecting the thermal stress curve with the strength curve [7].

As described above, the creep compliance, D(t), is an important rheological property of asphalt mixtures that constructs the relaxation modulus master curve of the TTSCM. Probably due to the effect of viscoplastic strain at high temperatures, the test temperatures for the creep test were lower in most cases (usually below 0 °C) [11,12,13]. Zofka et al. [14] investigated the creep compliance of twenty different asphalt mixtures at –6, −18, and −30 °C by the indirect tensile (IDT) test. Hill et al. [15] also used the IDT test to measure the creep compliance of asphalt mixtures, and the test temperatures were set to be 0, −12, and −24 °C. Moon et al. [8] performed the bending beam rheometer (BBR) test on an asphalt mixture to obtain its creep compliance at −24 and −12 °C, and then the TTSCM was performed to predict the cracking temperature. This leads to a lack of data on the relaxation modulus master curve at long reduced times, which may result in the mathematical model used for fitting the master curve showing abnormal trends due to extrapolation. This problem may cause errors in the predicted cracking temperatures of the TTSCM and therefore needs to be addressed or improved.

In order to quantify the degree of specific error of the predicted cracking temperature of the TTSCM, an experimental validation was needed. The thermal stress-restrained specimen test (TSRST) is the mainstream method for the prediction of low-temperature cracking [16]. In the TSRST, the asphalt mixture specimen is fixed, and thermal stress is generated by the contraction of the specimen owing to a drop in temperature. When the specimen is broken, the cracking temperature and failure are obtained. Although this test is time-consuming, it is close to the actual low-temperature cracking of asphalt pavements, and thus the test results, cracking temperature and failure stress, are relatively accurate [17,18]. Therefore, the TSRST can be considered as an effective experimental verification of the TTSCM.

In this study, the TTSCM was performed on styrene-butadiene-styrene (SBS) and base asphalt mixtures, and the prediction results were verified by the TSRST. Furthermore, an extended creep compliance test (ECCT) method, which adds creep compliance data at high temperature to modify the mathematical model, was developed to improve the TTSCM, and an in-depth analysis and recommendations for the ECCT were discussed. In this study, the prediction accuracy of the TTSCM was enhanced, and its range of applications was also extended.

## 2. Materials and Tests

### 2.1. Materials

**Figure 1:**
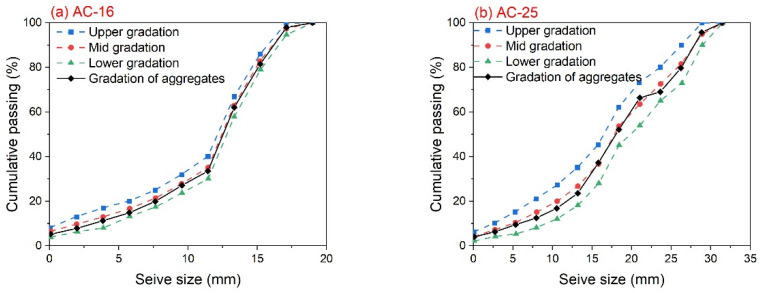
The gradation curves for (**a**) AC-16 and (**b**) AC-25.

In this study, two types of asphalt mixtures were considered: AC-16 and AC-25. Limestone was used as the aggregate, and the gradation curves for these two asphalt mixtures are depicted in Figure 1. The asphalt binders used for the AC-16 and AC-25 asphalt mixtures were SBS-modified asphalt and base asphalt, both sourced from Xinjiang, China. The properties of these asphalt binders are shown in Table 1 and Table 2. Using the Marshell test, the optimum asphalt contents of the AC-16 and AC-25 asphalt mixtures were determined to be 4.5% and 3.9%, respectively. The properties of these asphalt mixtures all satisfy the requirements of specification JTG F40-2004 [19], which is the common specification for asphalt pavements in China [20].

### 2.2. IDT Creep and Strength Test

**Figure 2:**
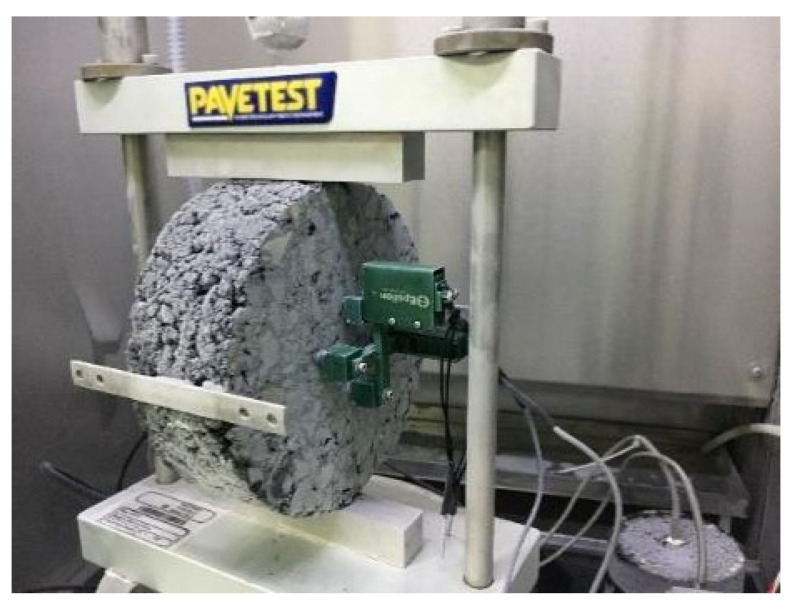
Test setup for IDT creep and strength tests.

IDT creep and strength tests were used to measure the creep compliance, D(t), and strength of the asphalt mixture, respectively, using a DTS-30 mechanical tester manufactured by Pavetest in Endeavour Hills, Australia. These two tests were conducted according to AASHTO T 322 specifications [21]. The test temperatures were −30, −20, and −10 °C for the creep test and −30, −20, −10, 0, and 20 °C for the strength test. Cylindrical specimens with dimensions of 150 mm diameter and 50 mm height were prepared for these tests, and each test temperature had three parallel specimens. However, for the strength tests under −30 and −20 °C, the load values for the specimen with a height of 50 mm exceeded 30 kN of the maximum load limit of DTS-30, and thus the height of the specimens was modified to 38 mm at these temperatures. The creep and strength test setup is shown in Figure 2. It can be seen that linear variable displacement transducers (LVDTs) were fixed in the center of the specimen, so as to measure the transverse and vertical displacements of the specimen, and both sides of the specimen are completely symmetrical. Before testing, the entire test setup needed to be placed in an environmental chamber for more than 4 h under test temperature.

For the creep test, the loading time was 1000 s, which ensured the applied load values changed in the range of ±2%, and the applied load values for the different asphalt mixtures at different temperatures were limited to ensure that the specimens remained in a linear viscoelastic state [15]. During testing, the transverse and vertical displacements of the specimen were automatically recorded, and the corresponding creep compliance values were calculated according to the method described by AASHTO T 322 [21]. To ensure the smoothness of the creep compliance curve, the creep compliance values at 1, 2, 5, 10, 20, 50, 100, 200, 500, and 1000 s were selected to construct the curve.

For the strength test, the specimen was loaded at a displacement loading rate of 12.5 mm/min until a decrease in the loading values occurred, and the strength of the asphalt mixture was calculated based on Equations (1) and (2) [21]:

(1) $$\begin{array}{c} S_{t,n}=\frac{2\times P_{f,n}}{\pi \times b_{n}\times {DI}_{n}}\times 0.001 \end{array}$$

(2) $$S_{t}=\frac{\sum_{n=1}^{3}S_{t,n}}{3}$$

(3) $$\begin{array}{c} C_{0}v=\frac{\sqrt{\frac{\sum_{n=1}^{3}{\left(s_{t,n}-S_{t}\right)}^{2}}{3}}}{s_{t}}\times 100 \end{array}$$

where $S_{t}$ is the average IDT strength of the asphalt mixture (MPa), $S_{t,n}$ is the IDT strength of the specimen (MPa), $P_{f,n}$ is the maximum loading value of the specimen (N), $b_{n}$ is the height of the specimen (mm), ${DI}_{n}$ is the diameter of the specimen (mm), and $C_{0}v$ is the coefficient of variation of parallel specimens (%).

### 2.3. Thermal Stress-Restrained Specimen Test (TSRST)

**Figure 3:**
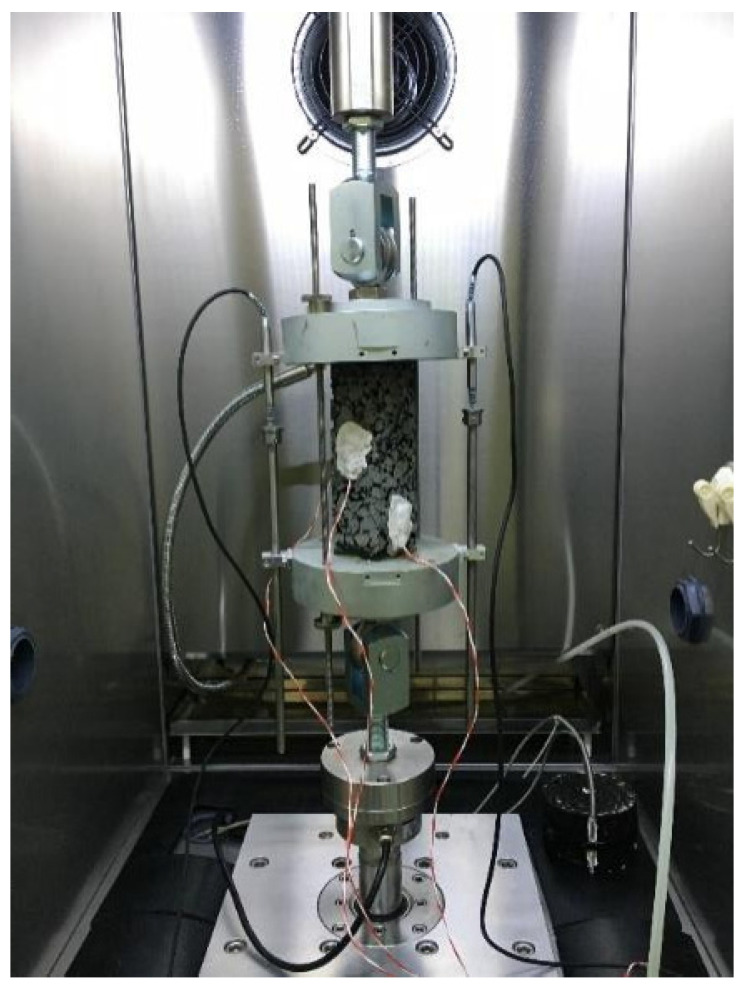
TSRST setup.

In this study, the TSRST was employed to verify the accuracy of the predicted cracking temperatures of the TTCSM for asphalt mixtures. The TSRST was conducted on a DTS-30 dynamic loading platform adhering to EN 12697-46 specifications [22]. The initial and final test temperatures were set to be 20 °C and −40 °C, respectively. Further, the cooling rate was set to be 10 °C/h. The tests used a 50 mm × 50 mm × 160 mm prismatic specimen, and each test had three replicates. The ends of the specimen were restrained to assure constant length during testing, and five temperature sensors were mounted at the top, center, and bottom of the specimen to acquire the temperature data of the specimen, as shown in Figure 3. In addition, the entire experimental setup was maintained in an environmental chamber under 20 °C for more than 4 h before testing. During testing, the thermal stress data with temperature were recorded automatically at intervals of 10 s until the specimen broke or the temperature reached −40 °C. The cracking temperature and failure stress of the asphalt mixture specimen are the temperature and stress at which the specimen breaks, respectively.

For the TSRST, the slope of the stress–temperature curve will become linear at lower temperatures, meaning that the asphalt mixture behaves as an elastic material [23]. According to the research of Stienss et al. [24], the temperature that separates the elastic region from the stress relaxation region of the asphalt mixture is defined as tangent point T_g_, which is around the starting temperature of 20 °C. Therefore, the effect of stress relaxation performance on the measured thermal stress results of TSRST can be ignored.

## 3. Thermal Stress Calculation Method

This study utilized the TTSCM to calculate the thermal stress in an asphalt mixture. First, the creep compliance, obtained by the laboratory creep test, was fitted as a power law function, $D\left(t\right)=D_{0}t^{n}$, where D(t) is the creep compliance (1/GPa), $D_{0}$ and n are both fitting parameters of the power law function, and t is the loading time (s), and was then converted into the relaxation modulus, E(t), using the following equations [25]:

(4) $$\begin{array}{c} \int_{0}^{\infty }D\left(t-\tau \right)\frac{dE\left(\tau \right)}{d\tau }d\tau =1 \end{array}$$

(5) $$\begin{array}{c} E\left(t\right)D\left(t\right)=\frac{\sin n\pi }{n\pi } \end{array}$$

where $D\left(t-\tau \right)$ is the creep compliance at time t−τ, 1/GPa; $E\left(\tau \right)$ is the relaxation modulus at time τ, GPa; n is the fitting parameter in power law function; and t is the loading time, s.

Subsequently, the relaxation modulus master curve was developed using the Williams–Landel–Ferry (WLF) model [26]:

(6) $$\begin{array}{c} \log\left(\alpha_{T}\right)=\frac{C_{1}\left(T-T_{0}\right)}{C_{2}+\left(T-T_{0}\right)} \end{array}$$

(7) $$\xi =\frac{t}{\alpha_{T}}$$

where $\alpha_{T}$ is shift factor; T is the test temperature, °C; T_0_ is the reference temperature corresponding to master curve, °C; C_1_ and C_2_ are the WLF parameters; ξ is the reduced time under reference temperature, s; and t is the loading time under test temperature, s. In this study, the reference temperature is set to be −30 °C.

For calculation convenience, regression analyses were conducted on the WLF model, and the regression equation is presented below [27]:

(8) $$\begin{array}{c} \xi =10^{a_{0}+a_{1}T+a_{2}T^{2}}t \end{array}$$

where ξ is the reduced time under −30 °C of reference temperature, s; T is the test temperature in °C; t is the loading time at test temperature, °C; and $a_{0}$, $a_{1}$, and $a_{2}$ are the regression parameters.

Once the relaxation modulus master curve is determined, it can be fitted using the generalized Maxwell (GM) model [28]:

(9) $$\begin{array}{c} E\left(t\right)=\sum_{i=1}^{n}E_{i}e^{-\frac{t}{\lambda_{i}}} \end{array}$$

where E(t) is the relaxation modulus at time t, MPa; $E_{i}$ is the elastic modulus corresponding to the ith spring, MPa; $\lambda_{i}$ is the viscosity coefficient corresponding to the ith damper, s; and t is the loading time, s.

Finally, the thermal stress was calculated using the following equation [7]:

(10) $$\begin{array}{c} \sigma \left(\xi \right)=\int_{0}^{\xi }\frac{d\varepsilon \left(\xi^{\prime }\right)}{d\xi^{\prime }}\cdot E\left(\xi -\xi^{\prime }\right)d\xi^{\prime }=\int_{0}^{t}\frac{d\left(\alpha \cdot \Delta T\right)}{dt^{\prime }}\cdot E\left(\xi \left(t\right)-\xi^{\prime }\left(t\right)\right)dt^{\prime } \end{array}$$

where $\sigma \left(\xi \right)$ is the thermal stress at time ξ; ξ is the reduced time under reference temperature, s; $E\left(\xi -\xi^{\prime }\right)$ is the relaxation modulus at time $\xi -\xi^{\prime }$; t is the loading time under test temperature; ΔT is the temperature difference at specific time intervals, associated with cooling rate [5], °C; and α is the thermal contraction coefficient, m/mm/°C. According to the research findings of Qadir [29], in this study the thermal contraction coefficients of AC-16 and AC-25 asphalt mixtures were assumed as 0.0000253 and 0.0000175 mm/mm/°C, respectively, based on their material properties, as described in Section 2.1.

By combining the GM model (Equation (8)), the final thermal stress calculation equation can be derived [8], as follows:

(11) $$\begin{array}{c} \sigma \left(t\right)=\sum_{i=1}^{N}\sigma_{i}\left(t\right) \end{array}$$

(12) $$\sigma_{i}\left(t\right)={\mathrm{e}}^{-\frac{\Delta \xi }{\lambda i}}\sigma_{i}\left(t-\Delta t\right)+{\alpha \Delta TE}_{i}\frac{\lambda_{i}}{\Delta \xi }\left(1-e^{-\frac{\Delta \xi }{\lambda_{i}}}\right)$$

where σ(t) is the thermal stress at time t, MPa; $\sigma_{i}\left(t\right)$ is the stress component corresponding to the ith Maxwell model at time t, MPa; Δt is the time intervals, s; Δξ is the reduced time intervals, s; and $E_{i}$ and $\lambda_{i}$ are both GM model parameters, as shown in Equation (9).

## 4. Original TTSCM Results

### 4.1. Creep Compliance

**Figure 4:**
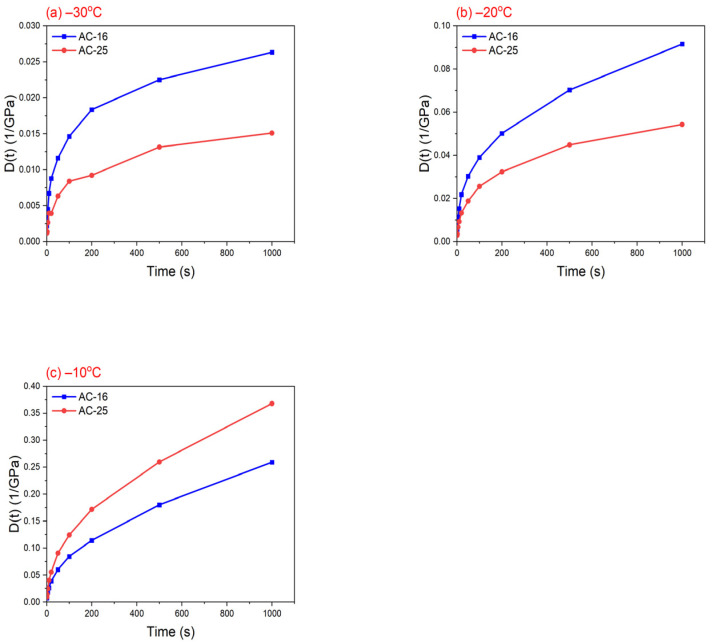
Creep compliance, D(t), at (**a**) −30 °C, (**b**) −20 °C, and (**c**) −10 °C for AC-16 and AC-25 asphalt mixtures.

Figure 4 illustrates the creep compliance curves for AC-16 and AC-25 asphalt mixtures at −30, −20, and −10 °C. It is worth noting that the coefficients of variation of creep compliance data for both AC-16 and AC-25 asphalt mixtures are all within 10%. The creep compliance, D(t), of these two asphalt mixtures increased with increasing loading time, whereas the rate of increase decreased. This trend is the same as that of the power-law function, $D\left(t\right)=D_{0}t^{n}$ (n < 1). Therefore, this study used a power-law function to fit the creep compliance curve, and the fitting results are listed in Table 3. It can be observed that the six sets of correlation coefficients, R^2^, are all higher than 0.97, implying high accuracy of the fitting results.

### 4.2. Relaxation Modulus Master Curves

**Figure 5:**
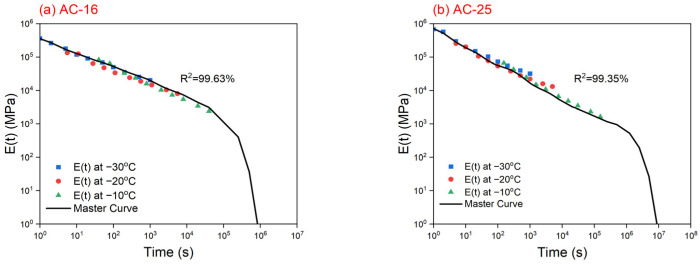
Relaxation modulus master curves for (**a**) AC-16 and (**b**) AC-25 asphalt mixtures.

Once the creep compliance, D(t), of these asphalt mixtures is determined, the relaxation modulus master curves at −30 °C of reference temperature can be calculated using the above-mentioned method, and the regression parameters of Equation (8) for AC-16 and AC-25 asphalt mixtures are presented in Table 4. For example, for AC-16 asphalt mixture under −10 °C, the creep compliance, D(t), at 100 s is 0.0845 (1/GPa), and the corresponding relaxation modulus, E(t), can be calculated by Equation (5), which is 7.3 GPa (7300 MPa). Further, the reduced time, ξ, can be determined by Equation (8), which is 3981.07 s. This implies that, for AC-16 asphalt mixture, the creep compliance, D(t), at 100 s under −10 °C corresponds to the point (3981.07, 7.3) at the relaxation modulus master curve. When the relaxation modulus master curves for the AC-16 and AC-25 asphalt mixtures were constructed, they were fitted using the GM model, and the results are presented in Figure 5 and Table 5. The correlation coefficients, R^2^, for these asphalt mixtures exceeded 0.99, guaranteeing the reliability of the fitting results.

### 4.3. Calculated Thermal Stress and Strength Curves

**Figure 6:**
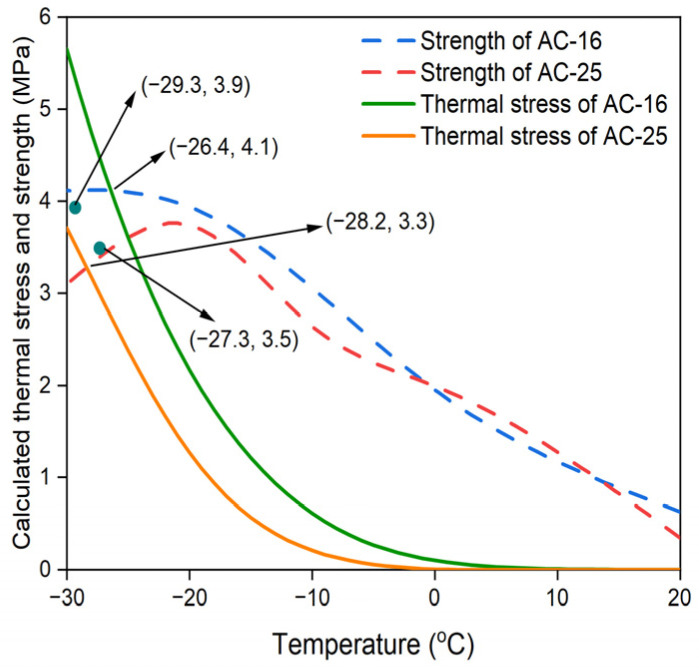
Calculated thermal stress and strength curves for AC-16 and AC-25 asphalt mixtures.

Furthermore, to compare with the test results of the TSRST, the initial temperature and cooling rate were set to be 20 °C and 10 °C/h, respectively, and the temperature difference, ΔT, at time intervals was set as 1 °C. Therefore, the time interval Δt was 360 s. Based on the above conditions, the thermal stresses for the AC-16 and AC-25 asphalt mixtures can be calculated using Equations (11) and (12). By combining the calculated thermal stress curves and the strength curves determined by the IDT strength test, whose coefficients of variation are all within 15%, the predicted cracking temperatures for these asphalt mixtures were determined, as depicted in Figure 6. It can be observed that the thermal stress of the AC-16 asphalt mixture was always higher than that of the AC-25 asphalt mixture under each temperature, and the predicted cracking temperature of the AC-16 and AC-25 asphalt mixtures were −26.4 °C and −28.2 °C, respectively. Furthermore, points (−29.3, 3.9) and (−27.3, 3.5) are the failure points for the AC-16 and AC-25 asphalt mixtures measured by TSRST, which will be described in Section 4.4.

### 4.4. TSRST Method

In this study, the measured cracking temperature and failure stress were defined as the average of corresponding measured values of three sets of parallel specimens for the TSRST, and the results are shown in Table 6. Figure 6 also depicts these measured failure points of AC-16 and AC-25 asphalt mixtures, and they are both close to respective strength curves, proving the reasonability of the results of the TSRST. Moreover, for the AC-16 asphalt mixture, the measured cracking point deviates significantly from the predicted cracking point, which means the original TTSCM prediction results may produce large errors for certain specific calculation conditions and materials.

### 4.5. Variance Analysis

The predicted and measured cracking temperatures obtained from the original TTSCM and TSRST are presented in Table 7. It can be observed that, for the AC-25 asphalt mixture, the predicted cracking temperature is only 0.9 °C (−3.1%) lower than the measured value, implying that the predicted result is relatively reasonable. However, for the AC-25 asphalt mixture, the predicted cracking temperature is 2.9 °C (−10.6%) higher than the measured value, indicating a serious error in prediction. To analyze the reasons for these results, the relaxation modulus master curves for both asphalt mixtures (Figure 5) is redisplayed. In this study, the reduced time obtained by interpolating the experimental data in the master curves is called the effective reduced time, and the effective reduced times for the AC-16 and AC-25 asphalt mixtures are within 39,600 s and 115,200 s, respectively. For both asphalt mixtures, as the reduced time exceeds the effective reduced time, owing to the extrapolation of data there is a sharp decline in the GM model until it is very close to zero, which does not conform to the common changing trend of the relaxation modulus of an asphalt mixture. Therefore, if a non-effective reduced time is used in the TTSCM, the predicted thermal stress may be erroneous, leading to an inaccurately predicted cracking temperature. For the AC-16 asphalt mixture, within the temperature range of −5 to 20 °C, the non-effective reduced times are used, which means the GM model cannot reflect all real relaxation modulus data, thus resulting in a higher predicted cracking temperature. For the AC-25 asphalt mixture, although the non-effective reduced times are used within the temperature range of 0 to 20 °C, the thermal stress was so small that it can be ignored in this temperature range, which means it is almost equivalent to the case where the initial temperature is 0 °C. Therefore, the predicted results for this asphalt mixture were relatively accurate.

## 5. Modified TTSCM Results

### 5.1. ECCT Method

**Figure 7:**
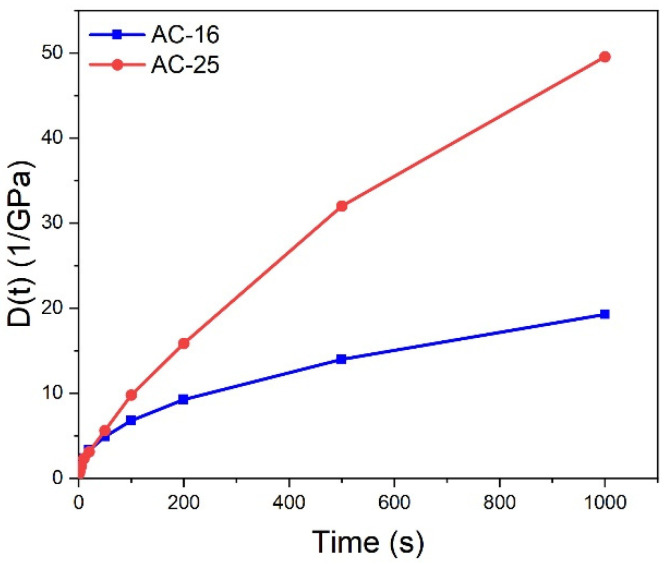
Creep compliance at 30 °C for AC-16 and AC-25 asphalt mixtures.

**Figure 8:**
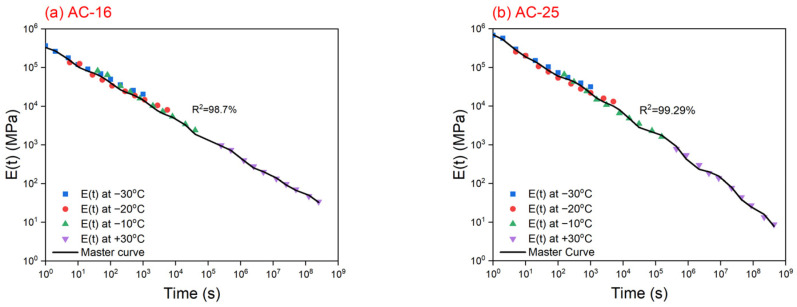
The modified relaxation modulus master curves for (**a**) AC-16 and (**b**) AC-25 asphalt mixtures.

To address the above problem, this study attempts to add creep compliance data at high temperatures to the relaxation modulus master curve to modify the GM model, which is called the ECCT method. Previous research [30,31,32,33] revealed that the testing temperature of the creep test should be no more than 30 °C to prevent the influence of viscoplastic strain, and therefore this study first selected 1000 s of creep compliance data at 30 °C to prolong the effective reduced time span of the GM model as much as possible. The reduced times of creep compliance at 1000 s under 30 °C are 1.42 × 10^9^ and 1.88 × 10^17^ s for the AC-16 and AC-25 asphalt mixtures, respectively, which can cover all reduced times in the thermal stress calculation process. The creep compliance curves and corresponding power law function fitting results for the AC-16 and AC-25 asphalt mixtures at 30 °C were determined using the IDT creep test, as shown in Figure 7 and Table 8, and the new master curves and the modified GM model parameters are presented in Figure 8 and Table 9. Similarly, the coefficients of variance of all creep compliance data at 30 °C are less than 10%. It can be observed that the correlation coefficients R^2^ of the GM model, incorporating the 30 °C curve, are slightly lower, registering at 98.7% for the AC-16 asphalt mixture and 99.29% for the AC-25 asphalt mixture, compared to the master curves excluding the 30 °C curve. This is due to the increase in the number of fitting data. However, by incorporating a 30 °C creep compliance curve, the sharp incline problem will not occur during short reduced times.

### 5.2. Modified Calculated Thermal Stress

**Figure 9:**
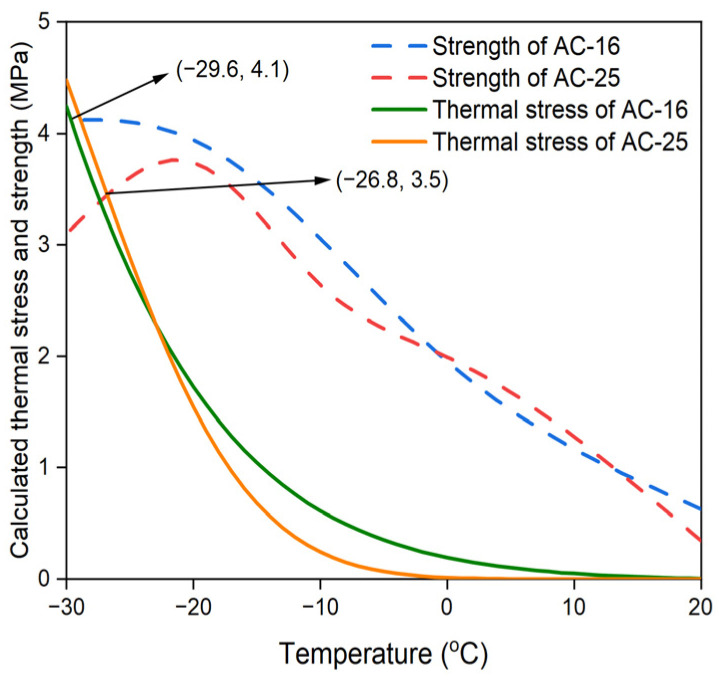
Modified calculated thermal stress and strength curves for AC-16 and AC-25 asphalt mixtures.

The modified thermal stress curves calculated using the modified GM model for the AC-16 and AC-25 asphalt mixtures are shown in Figure 9. Evidently, under less than −20 °C, the modified predicted thermal stresses of the AC-25 asphalt mixture are higher than those of the AC-16 asphalt mixture. Moreover, the modified predicted temperature of the AC-25 asphalt mixture is also higher than that of the AC-16 asphalt mixture, which is in good agreement with the results of the TSRST. For comparison, the modified predicted and measured temperatures obtained by the TSRST are listed in Table 10. It is clear that the modified predicted temperatures for the AC-16 and AC-25 asphalt mixtures are both acceptable, and are only 0.3 °C (−1.0%) lower and 0.5 °C (−1.8%) higher than the measured values, respectively. Therefore, the ECCT method can be considered effective for improving the TTSCM prediction accuracy of asphalt mixtures.

### 5.3. In-Depth Analysis

**Figure 10:**
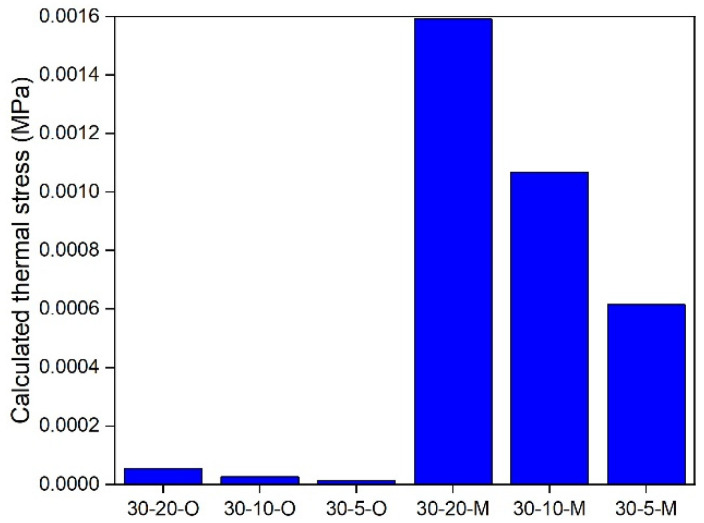
Calculated thermal stress under 30 °C.

**Figure 11:**
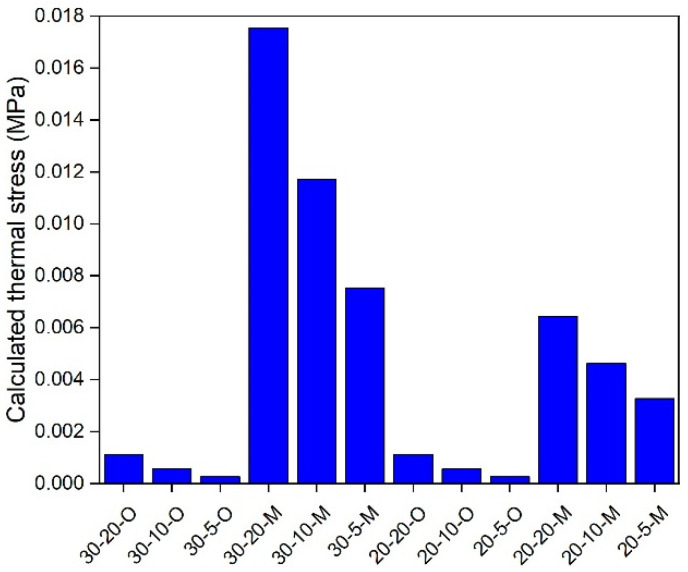
Calculated thermal stress under 20 °C.

**Figure 12:**
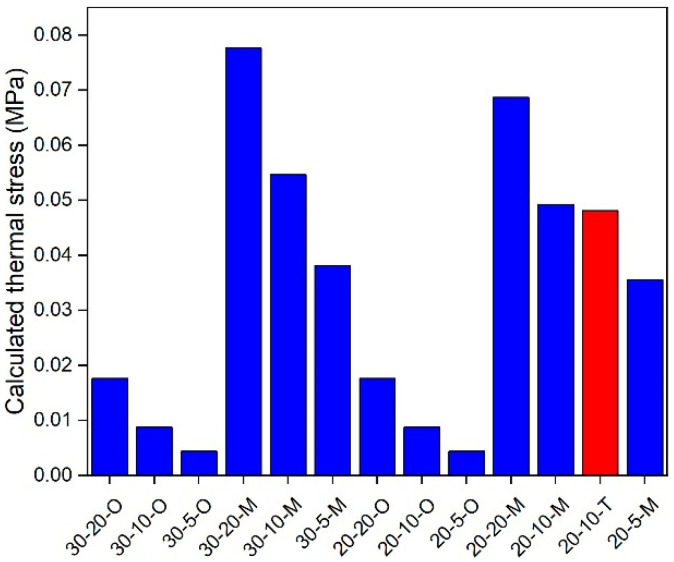
Calculated thermal stress under 10 °C.

**Figure 13:**
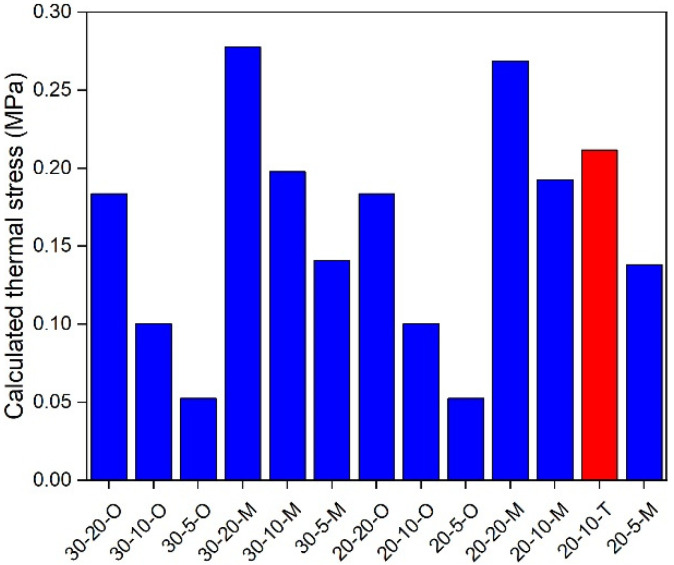
Calculated thermal stress under 0 °C.

**Figure 14:**
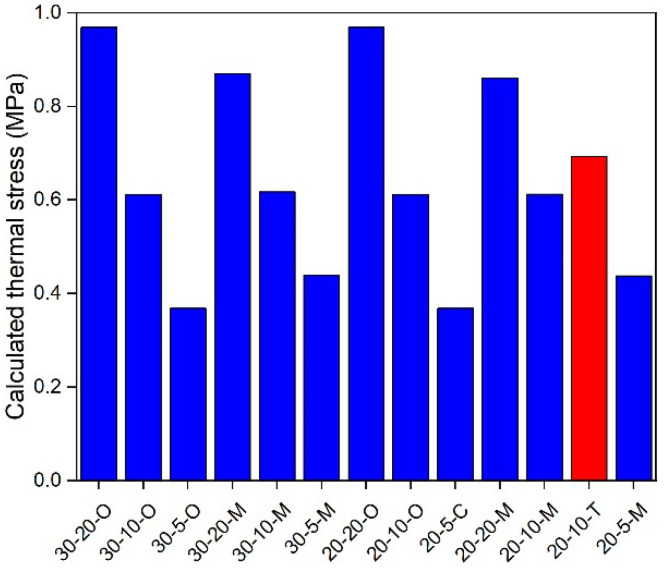
Calculated thermal stress under −10 °C.

**Figure 15:**
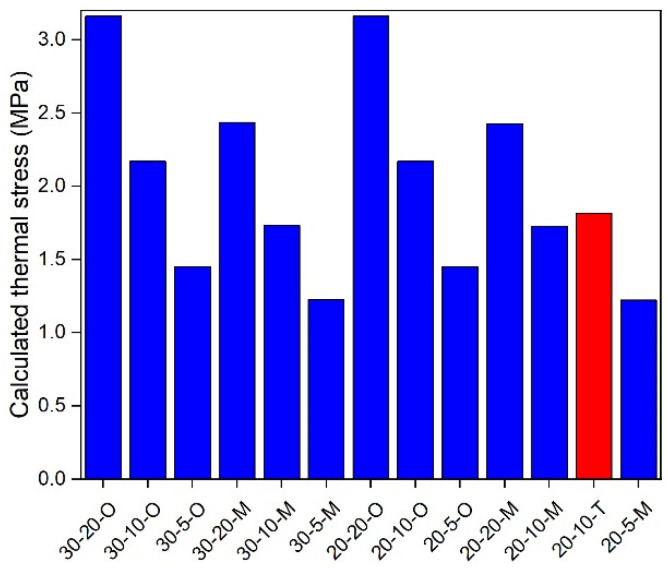
Calculated thermal stress under −20 °C.

**Figure 16:**
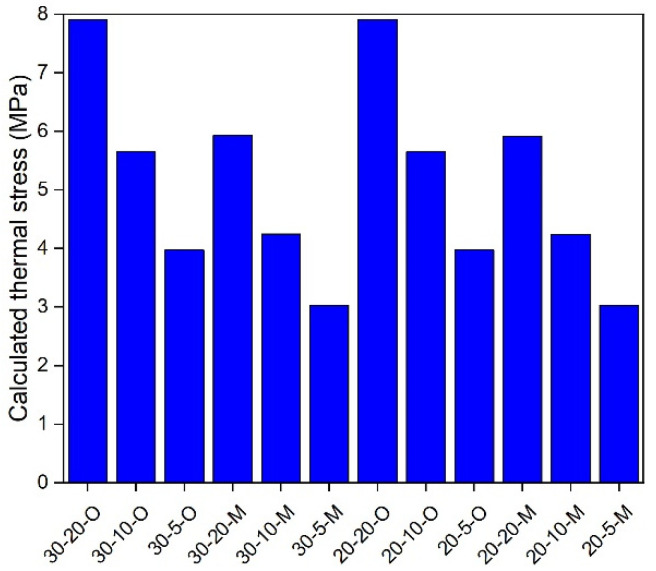
Calculated thermal stress under −30 °C.

An in-depth analysis of the modified and original TTSCM was employed to further understand the effects of the ECCT method. The thermal stress of an asphalt mixture is governed by both temperature and cooling rate [34]. Therefore, in this study, 5, 10, and 20 °C/h of cooling rates and 20 and 30 °C of initial temperatures were selected for a comparative analysis. Further, the temperature difference, ΔT, at specific time intervals is still set to be 1 °C, and thus the time intervals, Δt, for 5, 10, and 20 °C/h of cooling rates are 720, 360, and 180 s, respectively. Under these conditions, the effective reduced time span of the GM model modified by the ECCT method can cover all used reduced times. As the same thermal stress calculation method is used, this study only selected the AC-16 asphalt mixture for the analysis, and the results are shown in Figure 10, Figure 11, Figure 12, Figure 13, Figure 14, Figure 15 and Figure 16. In these plots, the calculation conditions are named in the form of the initial temperature–cooling rate–calculation method (O for the original TTSCM, M for the modified TTSCM, and T for the measured results by TSRST). It can be observed that at the same initial temperature and cooling rate, the thermal stresses of the modified calculation method are higher than those of the original calculation method at higher temperatures, which can be attributed to the fact that the ECCT increases the relaxation modulus, E(t), at non-effective reduced times, leading to higher thermal stress. However, at lower temperatures the thermal stresses of the original calculation method exceeded those of the modified calculation method, which can be attributed to the difference in the GM model parameters of the modified and original relaxation modulus master curves. The threshold is not constant but varies with the initial temperature and cooling rate. Moreover, the thermal stresses for both the modified and original calculation methods at the same temperatures increased with an increase in the initial temperatures. A plausible explanation for this is that, as the initial temperature increases, the gap between the initial and specific temperatures increases, resulting in the accumulation of greater thermal stresses. In addition, these thermal stresses increased with the rising of cooling rate, which is in line with the findings of Falchetto et al. [8]. In summary, the ECCT method can increase the calculated thermal stress values at higher temperatures and decrease them at lower temperatures but cannot change the basic changing pattern of the predicted thermal stress versus the initial temperature and cooling rate. Finally, at 10, 0, −10, and −20 °C, the measured thermal stresses by the TSRST (20-10-T) are all close to corresponding calculated values by the modified TTSCM (20-10-M), again proving the reliability of the ECCT method. 

### 5.4. Recommendation

Considering the influence of the viscoplastic strain in creep tests at high temperatures, the addition temperature of the ECCT is not recommended to be too high; however, it is suggested that it should be consistent with the initial temperature of the TTSCM. Moreover, for thermal stress calculation and cracking prediction of real asphalt pavement using TSRST, the initial temperature may not be constant. In this situation, the highest temperature of the region where the asphalt pavement is located is recommended as the addition temperature of the ECCT method. Further, the time span of the creep compliance data of the ECCT should be longer than the time intervals, Δt, of the TTSCM to ensure that the effective reduced time spans of the modified GM model can cover all used reduced times in thermal stress calculation. For example, creep compliance data over 360 s under 30 °C as the ECCT method is recommended for calculation conditions for 30 °C of initial temperature and 360 s of time intervals. If the initial temperature of the thermal stress calculation is higher than 30 °C, certain special calculation methods for the creep compliance of asphalt mixtures, such as creep compliances back-calculated from creep recovery data [35], should be used to obtain nearly linear viscoelastic data.

## 6. Conclusions

This study employed the TTSCM for AC-16 and AC-25 asphalt mixtures and verified the accuracy of the prediction results using the TSRST. In addition, the ECCT method was developed to enhance prediction accuracy. Finally, an in-depth analysis and recommendations of the ECCT method are discussed. The following conclusions were drawn from this study:

(1) For specific conditions and materials, the original TTSCM can produce inaccurate prediction results. For example, in this study the predicted cracking temperature of the AC-16 asphalt mixture using this method is −26.4 °C, which is 2.9 °C (−10.6%) higher than the measured value by the TSRST.
(2) This study developed an ECCT method by adding creep compliance data under high temperatures to the modified GM model to improve the prediction accuracy of the TTSCM. This study shows that the predicted cracking temperatures modified by the ECCT method for the AC-16 and AC-25 asphalt mixtures are both within ±2% error, implying a high accuracy.
(3) The ECCT method can increase the predicted thermal stresses at high temperatures, but it can decrease them at low temperatures. However, the predicted thermal stresses modified using this method conformed to a common changing pattern with respect to the initial temperature and cooling rate. Finally, it is recommended that the adding temperature of the extended relaxation modulus test be consistent with the initial temperature of the TTSCM, and the time span of creep compliance should be more than the time intervals of the TTSCM.

This study only set one condition, that is, an initial temperature of 20 °C and cooling rate of 10 °C/h for verification. Future studies should include a multiscale analysis of the calculated thermal stress data of the TTSCM based on various calculation conditions. Further, this study used only one mathematical model, the GM model, and thus, future research should verify the usability of the ECCT method on other mathematical models (such as the Christensen–Anderson–Marasteanu model [36]).

## Figures and Tables

**Table 1:** Properties of SBS-modified asphalt binder.

Properties	Unit	Tested Values	Requirements	Specification [19]
Penetration	0.1 mm	66	60–80	T0604
Penetration index	-	0.98	≥0.4	T0604
Ductility	cm	43	≥30	T0605
Softening point	°C	70.1	≥55	T0606

**Table 2:** Properties of base asphalt binder.

Properties	Unit	Tested Values	Requirements	Specification [19]
Penetration	0.1 mm	105	100–120	T0604
Penetration index	-	−1.31	−1.5–+1.0	T0604
Ductility	cm	80	≥40	T0605
Softening point	°C	46.6	≥43	T0606

**Table 3:** Power law function fitting results for AC-16 and AC-25 asphalt mixtures.

Materials	Temperature (°C)	Correlation Coefficient R^2^	n	D_0_ (1/GPa/s)
AC-16	−30	0.9816	0.3615	0.0026
	−20	0.9900	0.4197	0.0055
	−10	0.9972	0.515	0.0077
AC-25	−30	0.9948	0.3786	0.0013
	−20	0.9879	0.4385	0.0031
	−10	0.9749	0.53	0.0104

**Table 4:** Regression parameters for AC-16 and AC-25 asphalt mixtures.

Materials	a_0_	a_1_	a_2_
AC-16	2.5714821	0.102559929	0.0005614
AC-25	4.113412	0.23789485	0.0033592

**Table 5:** GM model parameters for AC-16 and AC-25 asphalt mixtures.

AC-16		AC-25	
$E_{i}$(MPa)	$\lambda_{i}$(s)	$E_{i}$(MPa)	$\lambda_{i}$(s)
4487.86	104,000	1409.64	1,260,000
8861.79	7880	2948.62	41,600
310,711.91	1.65	755,721.20	2.01
25,423.99	830	11,315.63	4020
104,341.69	10.8	193,891.60	20.8
53,159.19	87.2	51,050.31	375

**Table 6:** The measured results of TSRST for AC-16 and AC-25 asphalt mixtures.

Materials	Cracking Temperature (°C)	Failure Stress (MPa)
AC-16	−29.3	3.9
AC-25	−27.3	3.5

**Table 7:** The predicted and measured cracking temperatures for AC-16 and AC-25 asphalt mixtures.

Materials	Predicted Cracking Temperatures (°C)	Measured Cracking Temperatures (°C)
AC-16	−26.4	−29.3
AC-25	−28.2	−27.3

**Table 8:** The power law function fitting results for AC-16 and AC-25 asphalt mixtures at 30 °C.

Materials	Temperature (°C)	Correlation Coefficient R^2^	n	D_0_ (1/GPa/s)
AC-16	+30	0.9967	0.4854	0.7118
AC-25	+30	0.997	0.6548	0.4994

**Table 9:** The modified GM model parameters for AC-16 and AC-25 asphalt mixtures.

AC-16		AC-25	
$E_{i}$(MPa)	$\lambda_{i}$(s)	$E_{i}$(MPa)	$\lambda_{i}$ (s)
76.31846996	296,697,543	32.152611	307,446,242.6
196.0472279	11,514,043	229.51479	14,033,219.23
310,194.5409	3.3348825	748,302.48	2.176162918
998.7223319	641,250.84	2370.6892	337,049.4659
20,310.38025	974.19383	49,870.711	511.9427406
6080.484499	18,160.205	13,141.398	8743.14716
71,984.08669	62.080632	174,116.97	24.20942642

**Table 10:** The modified predicted and measured cracking temperatures for AC-16 and AC-25 asphalt mixtures.

Materials	Modified Predicted Cracking Temperatures (°C)	Measured Cracking Temperatures (°C)
AC-16	−29.6	−29.3
AC-25	−26.8	−27.3

## Data Availability

The original contributions presented in the study are included in the article, further inquiries can be directed to the corresponding author.
